# Evaluation of smartphone interactions on drivers’ brain function and vehicle control in an immersive simulated environment

**DOI:** 10.1038/s41598-021-81208-5

**Published:** 2021-01-21

**Authors:** Joseph M. Baker, Jennifer L. Bruno, Aaron Piccirilli, Andrew Gundran, Lene K. Harbott, David. M. Sirkin, Matthew Marzelli, S. M. Hadi Hosseini, Allan L. Reiss

**Affiliations:** 1 Division of Brain Sciences, Department of Psychiatry and Behavioral Sciences, Center for Interdisciplinary Brain Sciences Research, School of Medicine, Stanford University, 401 Quarry Rd., Stanford, CA 94305 USA; 2 Department of Mechanical Engineering, Stanford University, Stanford, CA 94305 USA; 3 Department of Radiology, Stanford University, Stanford, CA 94305 USA

**Keywords:** Attention, Cognitive control, Decision, Problem solving

## Abstract

Smartphones and other modern technologies have introduced multiple new forms of distraction that color the modern driving experience. While many smartphone functions aim to improve driving by providing the driver with real-time navigation and traffic updates, others, such as texting, are not compatible with driving and are often the cause of accidents. Because both functions elicit driver attention, an outstanding question is the degree to which drivers’ naturalistic interactions with navigation and texting applications differ in regard to brain and behavioral indices of distracted driving. Here, we employed functional near-infrared spectroscopy to examine the cortical activity that occurs under parametrically increasing levels of smartphone distraction during naturalistic driving. Our results highlight a significant increase in bilateral prefrontal and parietal cortical activity that occurs in response to increasingly greater levels of smartphone distraction that, in turn, predicts changes in common indices of vehicle control.

## Introduction

Driving an automobile is a common daily experience for millions of people around the world. Despite the challenges facing each driver, which include but are not limited to visual and auditory distractions that divert attention from the road, the majority of these daily automobile trips are successful and do not end in injury or death. When accidents do occur, they are often due to driver distraction^1^. In the absence of other forms of driver impairment (e.g., drug/alcohol use, drowsiness, or emotional agitation), driver distraction is a critical factor in more than 50% of crashes that result in injury or property damage in the US^2^. According to the US National Highway Traffic Safety Administration, in 2015 a total of 3,447 people were killed, and 391,000 injured, in vehicle accidents involving a distracted driver^3^. In 2016 that number rose to approximately 3,765 distraction-related driving fatalities, making up approximately 9.2% of total fatalities and marking an increase of 8% since 2014^4^.

Among the many distractions a driver is likely to encounter is the smartphone^1,4^. According to the Pew Research Center, in 2018 approximately 95% of Americans owned a cellphone of some type, and 77% owned a smartphone. A meta-analysis of naturalistic driving studies concluded that texting or browsing on a smartphone increased the odds of a safety–critical event while driving by more than tenfold^5^. Nevertheless, despite classifying texting as one of the most dangerous tasks to do while driving (second only to driving drunk), 51% of drivers reported interacting (i.e., sending/receiving texts, navigation) with their phone while driving^6^. This is particularly troubling, as drivers distracted by a secondary task contributed to over 22% of all crashes and near-crashes, with over one third of these secondary tasks involving interaction with a wireless device^7,8^. This problem is compounded by the ever-growing ubiquity of smartphone apps, many of which are designed to enhance the driving experience^9^, but which inherently require driver attention^4^. Indeed, studies have shown that interaction with GPS and other driver-assistance apps often leads to distraction and impairments in driving performance^10^.

An important component to understanding, and thus possibly improving, driver safety is the elucidation of the brain and behavioral signatures that underlie naturalistic driving. To that end, previous research using fMRI has identified the engagement of a network of brain regions including the motor, parietal, occipital, prefrontal, and cerebellar cortices that are commonly elicited during driving tasks^11–19^. Common driving maneuvers such as turning, reversing, stopping, and monitoring other cars and traffic rules have all been shown to elicit activity from these regions^16,17^. Notably, distraction in the form of a hands-free phone conversation while turning elicits a predictable shift in neural activity to the prefrontal cortex, thus highlighting a possible neurobiological signature of distraction while driving^16^.

While providing much needed insight into the brain’s response to safe as well as distracted driving, methodological constraints inherent in fMRI limit the generalizability of these findings with regard to real world driving. Specifically, the experience of lying supine in an MRI bore while interacting with a simulated vehicle in a manner that is unlike naturalistic driving may elicit patterns of neural activations that do not occur in real world driving. Moreover, because movement is severely restricted in fMRI studies, the ability to interact with a smartphone as one normally would is not possible.

As an alternative to fMRI, previous research using EEG in a simulated driving environment has demonstrated changes in prefrontal and parietal activity in response to increased cognitive workload^20–22^. Using a battery of common in-vehicle activities including listening to music, conversing with friends, or using a speech-to-text email service, Strayer and colleagues^22^ identified electrocortical signatures of cognitive workload that coincided with attentional demands of the task. Similarly, Lei and Roetting^20^ employed a combination of lane change maneuvers and an n-back task to demonstrate changes in alpha and theta signatures in response to increased cognitive workload.

Clearly, the use of EEG in driving research has multiple benefits over fMRI, particularly regarding its amenability to more naturalistic environments and tasks. However, similar to fMRI, EEG often suffers from methodological drawbacks, primarily related to motion artifacts^23^ and environmental noise^24^, that hinder its use in studies of real-world driving. Moreover, the poor spatial resolution of EEG^25^ often makes localization of cortical activity difficult, thus further reducing its ability to highlight specific cortical regions that underlie common driving behaviors. Thus, while significant steps have been taken, little is known regarding the neurobiological signatures of distraction while engaging in naturalistic driving. We argue that functional near-infrared spectroscopy (fNIRS) affords a light-weight and portable brain imaging modality that is less sensitive to movement, thus making it amenable to driving studies^26–29^.

**Figure 1:**
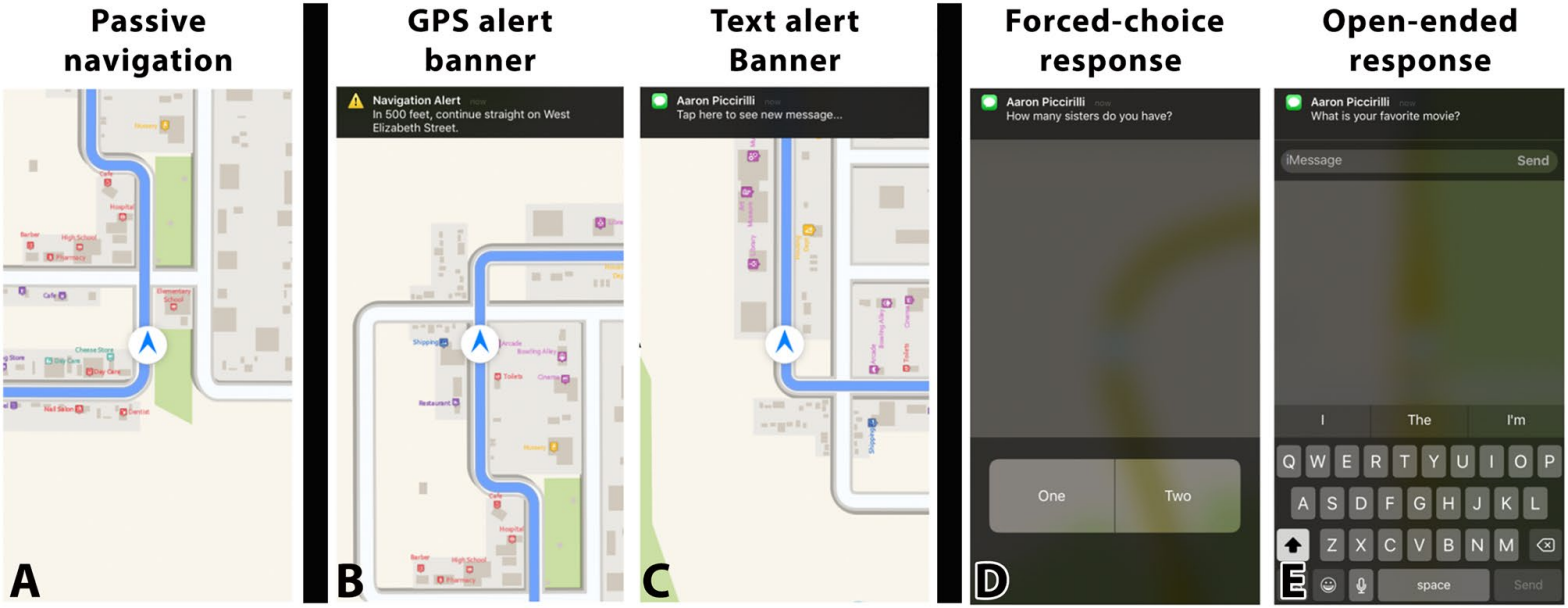
Smartphone visual displays. Participant’s experienced each form of visual feedback shown above. (**A**) The passive GPS display provided a continuously updated map displaying a topographical layout of the driver’s surroundings, an overlay of the route the driver was meant to follow, and a blue chevron representing the driver’s position. (**B**) GPS alerts were presented via a black banner across the top of the passive GPS display. (**C**) Identical alert banners preceded both text response conditions and were accompanied by a ‘ding’ sound. Participants were instructed to press the text alert banner to initiate their response action at their earliest safe convenience. (**D**) Within a forced-choice text response (FCR), participants were instructed to press one of two buttons to indicate their response to the message presented in the text alert banner. One of the two buttons contained a correct response option that was drawn from their survey responses. The alternate button contained an incorrect but plausible response. (**E**) Within an open-ended text response (OER), participants were instructed to type their response as they normally would on a standard QWERTY keyboard. All typed text appeared in a text display field directly under the text alert banner. After a response was typed, participants pressed a ‘Send’ button located directly to the right of the text display field. The text response display was removed when the ‘Send’ button was pressed.

Here, we employed fNIRS to examine cortical activity in response to smartphone distractions within an immersive, full-cab driving simulator. In our study, participants drove a predetermined course while receiving GPS navigation alerts and text messages. Specifically, five conditions were established that provided the driver with differential levels of distraction, thus capturing the wide range of disruptions that often occur in the real world (see Fig. 1 for example displays of each condition; see Smartphone Display and Alerts section below for a description of each distraction type).

**Figure 2:**
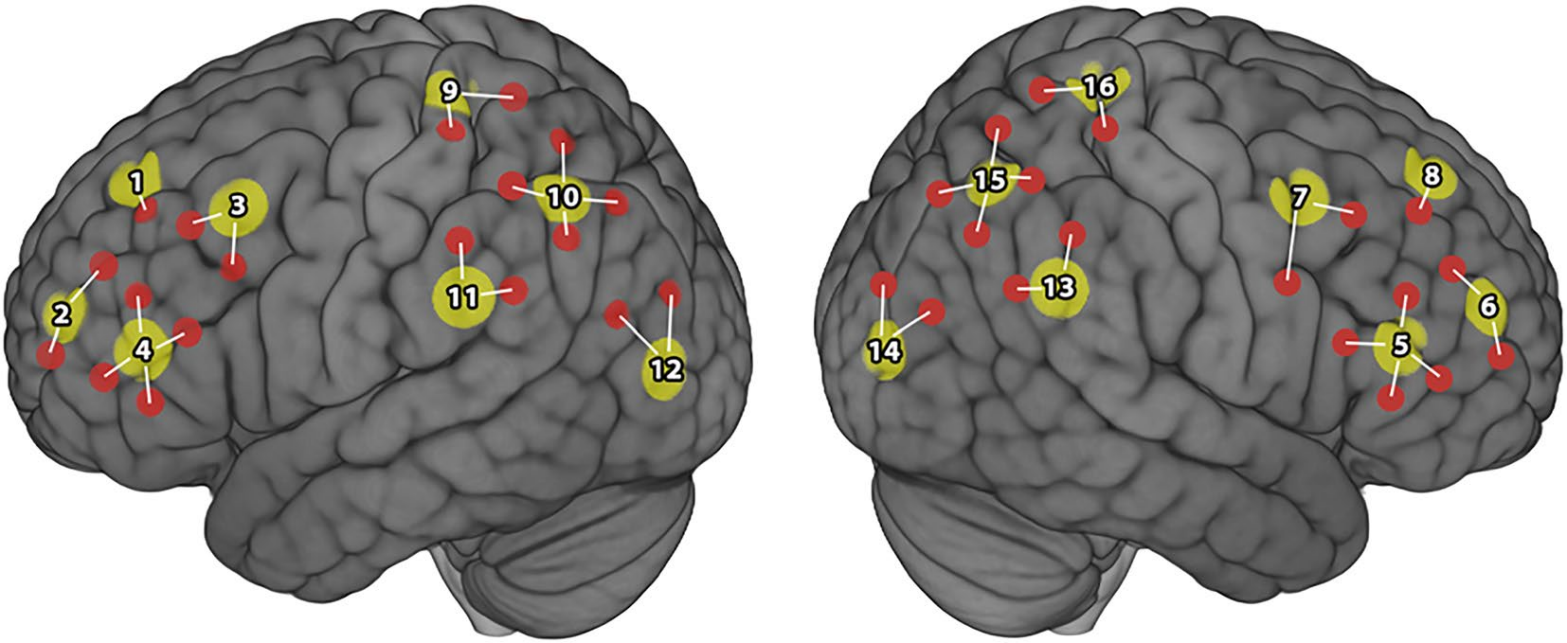
Optode configuration and channel clustering. A total of 16 source (yellow dots) and detector pairs were situated over the bilateral prefrontal and parietal cortices to form a total of 38 recording channels (red dots). We employed a source channel clustering, wherein each channel associated with a given source were clustered together to form a region of interest (N = 16). For each participant, the single channel within each cluster that responded greatest to OER vs. CD contrasts was selected as their responding channel and were used for each analysis. The source clusters are associated with the following anatomical regions: S1 = left superior frontal pole; S2 = left inferior frontal pole; S3 = left dorsolateral prefrontal; S4 = left ventrolateral prefrontal; S5 = right ventrolateral prefrontal; S6 = right inferior frontal pole; S7 = right dorsolateral prefrontal; S8 = right superior frontal pole; S9 = left superior parietal; S10 = left central parietal; S11 = left angular gyrus; S12 = left temporo-parietal junction; S13 = right angular gyrus; S14 = right temporo-parietal junction; S15 = right central parietal; S16 = right superior parietal.

Throughout the drive, we recorded concurrent measurements of participants’ cortical activations in the bilateral parietal and prefrontal cortices, patterns of eye gaze, and driving behavior (see Table 1 for description of driving behavior metrics). As stated above, the bilateral parietal and prefrontal cortices have been implicated in driving tasks and are thus regions of interest for our study^11–19^. Specifically, the regions of interest assessed included the bilateral superior frontal poles, inferior frontal poles, dorsolateral prefrontal cortices, ventrolateral prefrontal cortices, superior parietal, central parietal, angular gyrus, and temporo-parietal junction (see Fig. 2 for fNIRS optode configuration and channel clustering). The behavioral metrics outlined in Table 1 are associated with lateral and longitudinal vehicle control, which are significant predictors of driving performance^30,31^.

**Table 1:** Descriptions of behavioral metrics.

Metric	Definition
Lateral acceleration^a^	Rate of change of the vehicle’s velocity in the lateral direction, measured in m/s^2^
Lateral jerk^b^	Rate of change of lateral acceleration of the vehicle in m/s^3^
Heading error^c^	Angle (in degrees) between the road path and the heading of the vehicle
Lane excursion duration^d^	Time, in seconds, during which the vehicle occupied two lanes simultaneously
Lane drift^e^	Summation of all lateral movement of the vehicle
Steering wheel angle^f^	Angle of the steering wheel in degrees
Steering wheel reversals^g^	Quantification of all changes in direction of the steering wheel turn (i.e., change in the sign of the steering angle)
Brake pedal force^h^	Force, in Newtons, applied to the brake pedal

The metrics above were used to capture the behavior of the car and driver throughout the duration of the study. ^a–e^Provide metrics of how the car advanced through the virtual course, and ^f–h^provide metrics of how the driver interfaced with the car via the steering wheel and brake.

We hypothesize that increasing levels of smartphone distraction will coincide with changes in driver’s brain and behavior. Specifically, we hypothesize that increased attentional demands resulting from reading and responding to text messages will result in greater cortical activity, particularly in the prefrontal cortices of interest (see Fig. 2). Relatedly, we expect greater attentional demands from smartphone interactions to result in increased behavioral driving metrics (e.g., lateral acceleration) that are often associated with poor driving performance. Finally, we expect to identify significant predictive relationship between cortical activation and driving behavior, as well as between cortical activation and sympathetic nervous system responses (i.e., pupil dilation). We hypothesize that the identification of such changes will highlight new and important neurobiological signatures of distracted driving.

## Materials and methods

### Participants

A total of 26 (N_female_ = 14) participants were recruited for participation. Data from four participants were discarded due to technical issues during data collection, and an additional five were lost due to illness developed while driving the simulator. A total sample size of 17 (N_female_ = 11) was included in the final data set. A sample size analysis based on previously recorded data (Cohen’s *d* = 0.78) indicated that an N of 15, given a power of 0.8 and an alpha of 0.05, is suitable for detecting a significant group-level fNIRS contrast effect. Given the difficulty of our study design and expected attrition due to simulator sickness, we recruited greater than N = 15 participants. The Stanford University Institutional Review Board approved all study procedures including informed consent and experimental protocols. Written informed consent was obtained from the participants prior to participation. Moreover, all activities related to this experiment were performed in accordance with relevant guidelines and regulations.

### Car simulator and environment

**Figure 3:**
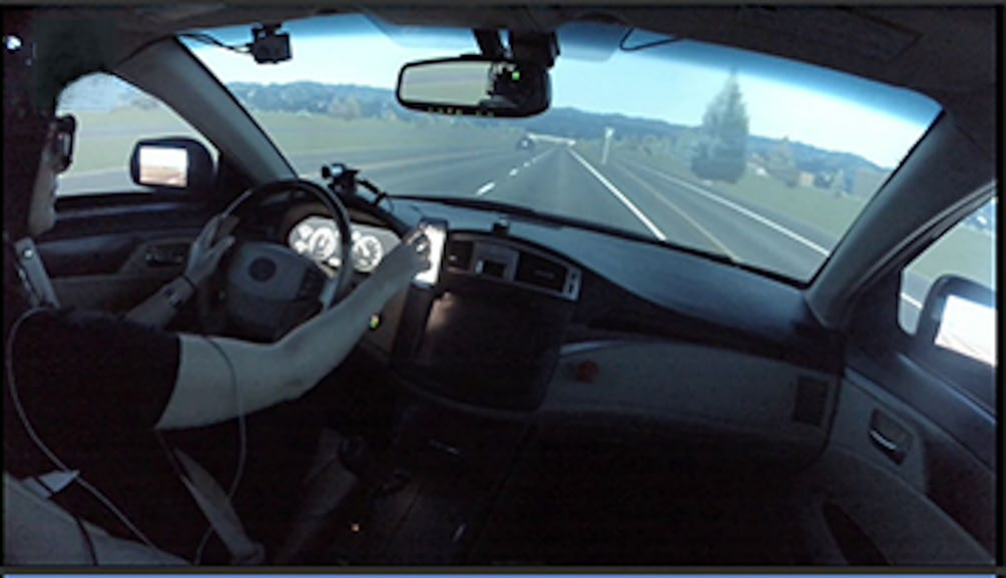
In-cabin view of the driver and their surroundings. Participant’s experienced a 270° field of view of the driving environment, including rear projection for the rear-view mirror and LCD-screen side mirrors. The smartphone was attached to a standard aftermarket magnetic dash mount.

In order to assess the impact of drivers’ interactions with distracting technologies during naturalistic yet safe driving, we employed an immersive, fixed-base, full-cab driving simulator (Realtime Technologies Inc., United States) that was identical to that reported previously^26^. The simulator consisted of a full vehicle cab (Toyota Avalon) with LCD-screen instrument cluster and center stack panels, high-resolution 270° field-of-view cylindrical projection screen, rear projector for the rear-view mirror, and LCD-screen side mirrors (Fig. 3). Images from 5 projectors were blended onto the cylindrical screen to form the forward and peripheral visual environment. The steering wheel provided force feedback that emulated normal driving forces, and road and engine noises were provided by a customized surround system.

**Figure 4:**
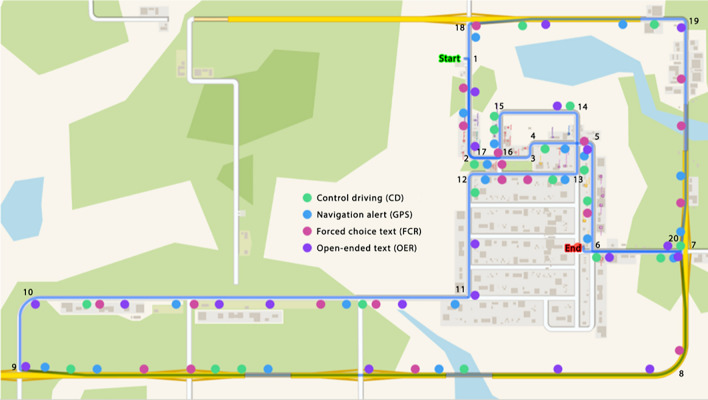
Birds-eye view of the simulated environment and driving route. Each participant followed the same pseudorandom route, which is given by the Arabic numerals placed along the blue driving path. The numbers are placed at each 90° turn, beginning from the ‘Start’ location and ending at the ‘End’ location. Each dot represents the location at which an event was initiated. The event order was arranged so that each event could be completed prior to the successive event beginning. The alert banners for the FCR and OER conditions were identical. The CD events did not initiate any change in the smartphone display and were thus invisible to the driver.

The simulated environment was developed in Internet Scene Assembler^32^ and saved using Virtual Reality Modeling Language (VRML). The Internet Scene Assembler facilitates the creation of interactive and dynamic 3D scenes that involve complicated logic and behavior. Each participant traveled in a pre-determined route throughout the simulated environment (see Fig. 4). The environment contained a mixture of city and highway landscapes, which provided context-specific driving challenges (e.g., more frequent stops in the city) and conditions (e.g., higher speed limit on highways). The order and location of each smartphone event (see Smartphone display and alerts section below) was pseudo-randomized prior to data collection. The timing of each event was situated throughout the environment to avoid overlap. Event markers based on position in the course were used to timestamp data and link fNIRS, eye tracking and smartphone data streams. The simulator software recorded multiple aspects of the drivers’ behavior at a sampling rate of 60 Hz as they navigated through the environment (see Table 1). As shown in Table 1, eight behavioral metrics were used to capture a range of variables that describe how the car advanced through the virtual course (Table 1^a–e^), as well as how the driver interfaced with the car via the steering wheel and brake in order to complete the virtual course (Table 1^f–h^). While many of these variables overlap (e.g., steering wheel angle vs. steering while reversals), each provides unique information about how the driver navigated the course, and may be expected to interact with drivers’ neural, physiological, or neuropsychological signatures.

Prior to beginning the study, participants were required to complete a short drive in the simulator. This allowed participants to acclimate themselves to the simulator dynamics and visual elements of the environment, as well as to ensure that each participant experienced an equal amount of training prior to the study. Furthermore, this practice drive served to identify participants who experienced simulator sickness and thus could not participate in the remainder of the study. Simulator sickness is a syndrome similar to motion sickness, often experienced during simulator or virtual reality exposure^33^. A total of five participants experienced significant simulator sickness and chose to end their participation in the study.

### Smartphone display and alerts

The visual displays of all smartphone interfaces and alerts were designed to mimic iOS 11, and an iPhone 6 was used for all participants. In order to ensure that all participants were familiar with this interface, current use of an iPhone was an inclusion criterion. Prior to driving, all participants completed a general information survey about themselves (e.g., number of family members, favorite food, etc.). The smartphone was held securely to the dashboard of the car via a standard aftermarket magnetic dash mount (see Fig. 3). Participants were encouraged to hold and interact with the phone as they normally would (e.g., remove it from the mount to type). During each participant’s drive, questions relating to their survey responses were sent as text messages.

In total, participants experienced five unique smartphone displays (see Fig. 1 for example visual displays): Passive GPS display: including a passive GPS display, GPS alerts, text alerts, forced-choice text response screens, and open-ended text response screens.

- Passive GPS: The passive GPS display provided a continuously updated map displaying a topographical layout of the driver’s surroundings, an overlay of the route the driver was meant to follow, and a blue chevron representing the driver’s position. The passive GPS display was always present and served as a natural control condition. In order to maintain statistical power for comparisons with the other conditions, we included 20 passive GPS event markers in our data stream.
- GPS alerts: GPS alerts were presented via a black banner across the top of the passive GPS display. The onset of each banner was paired with an auditory alert, and the GPS banner remained present on the display for 7 s. A total of 20 GPS alert banners were presented throughout the course.
- Text alerts: Identical alert banners preceded both text response conditions and were accompanied by a ‘ding’ sound. Participants were instructed to press the text alert banner to initiate their response action at their earliest safe convenience. Each text alert banner remained present on the display for a maximum of 20 s. Upon pressing the text alert banner, participants were presented with either a forced-choice or open-ended response option. In both cases, the passive GPS was overlaid with the text response display. A total of 40 text alert banners were presented.
- Forced-choice text response screens: Within a forced-choice text response (FCR), participants were instructed to press one of two buttons to indicate their response to the message presented in the text alert banner. One of the two buttons contained a correct response option that was drawn from their survey responses. The alternate button contained an incorrect but plausible response. The text response display disappeared and was replaced with the passive GPS display immediately after a response was made. Participant’s had a maximum of 12 s to respond from the time they pressed the text alert banner, after which the text response display was removed. A total of 20 forced-choice text response events were presented.
- Open-ended text response screens: Within an open-ended text response (OER), participants were instructed to type their response as they normally would on a standard QWERTY keyboard. All typed text appeared in a text display field directly under the text alert banner. After a response was typed, participants pressed a ‘Send’ button located directly to the right of the text display field. The text response display was removed when the ‘Send’ button was pressed. Participants were given 30 s to respond from the time they pressed the text alert banner. A total of 20 open-ended text response events were presented.

### Biological monitoring tools

#### fNIRS

A tandem NIRScoutX (NIRx, Germany) system was used to record hemodynamic responses using two wavelengths (760 and 850 nm) with 16 LED illumination time-multiplexed sources and 16 avalanche photodiode sensors, sampling at a frequency of 7.8125 Hz. The optodes were positioned over the bilateral PFC and bilateral parietal cortices (see Fig. 2). Optodes were positioned over standard 10–20 system locations using individually sized caps (Brain Products, Germany) to maintain consistency across variations in head sizes^34,35^. Plastic supports were placed between each source/detector pair that constituted a recording channel to maintain a 3 cm source-detector distance. This consistency allowed us to subset the fNIRS channels of interest down to those directly measuring each region of interest, and to cluster those channels using established methods^26,36,37^.

#### Eye tracking

SMI ETG 2.0 binocular eye tracking goggles (SensoMotoric Instruments, Germany) were used to measure the gaze patterns of participants during each distracting event. The goggles use two infrared cameras (one for each eye) integrated in the inner eyeglass frame to capture eye movement at a sampling rate of 60 Hz.

#### Self-report inventories

Each participant completed self-report versions of the Neuroticism-Extroversion-Openness Five-Factor Inventory (NEO-FFI) personality^38^ and the Behavior Rating Inventory of Executive Function (BRIEF)^39^ to examine the potential relationships between personality factors/executive functioning and vehicle control, cortical activation, and eye gaze patterns. Inclusion of these inventories allowed for us to test a priori hypotheses that personality characteristics relate significantly to driving behavior.

### Data analysis

#### fNIRS analyses

Prior to analysis all fNIRS data were pre-processed using Matlab-based functions derived from Homer2. First, the raw optical density data was motion corrected using a wavelet-based motion artifact removal process^40^. Next, the motion corrected data were band-pass filtered using the low- and high-pass parameters of 0.5 and 0.01 respectively. This filtered optical density data were then converted to oxy-hemoglobin (HbO) and deoxy-hemoglobin (HbR) values by way of the modified Beers-Lambert law. We employed a generalized linear model (GLM) approach to analyze our fNIRS data^26,36,41,42^. Specifically, a separate GLM was used to quantify cortical activations associated with HbO and HbR concentration for each analysis or condition listed below^43,44^. We employed an fNIRS source-based channel clustering and functional localization data reduction approach similar to those reported previously^26,36,37^. Channel localization within each cluster was first established within the HbO data, and then assessed within the same channels for the HbR data. Only those clusters that were significant in both data sources were reported below. All visualizations were based on HbO data.

Our first GLM employed parametric modulation of the amplitude of the convolved hemodynamic response function to each condition based on a priori classification of condition-related distraction. As shown in Fig. 1, all 5 conditions were categorized ordinally from least to most distracting. This approach allowed us to identify regions of the cortex whose activity increased linearly as participants engaged in increasingly distracting tasks. Conversely, this approach also allowed for the identification of regions whose activity may be higher in lesser distracting tasks, and which decreased as our operational definition of distraction increased. Second, we employed a standard GLM condition contrast approach that modeled each condition individually. The resulting t-values associated with each condition’s beta estimation were submitted to a priori condition contrasts, and the significance of the contrast outcomes were assessed via one-sample t-tests.

#### Behavioral metric analysis

Table 1 provides a brief definition of each vehicle control metric of interest. Condition-wise mean values were calculated based on all observations made throughout the entirety of each event. Identical one-way repeated measures ANOVA’s were conducted to compare each behavioral metric between five conditions. Next, follow-up comparisons were made between each condition. All follow-up comparisons were corrected for inflated Type I error using the FDR correction method.

#### Eye tracking analysis

**Figure 5:**
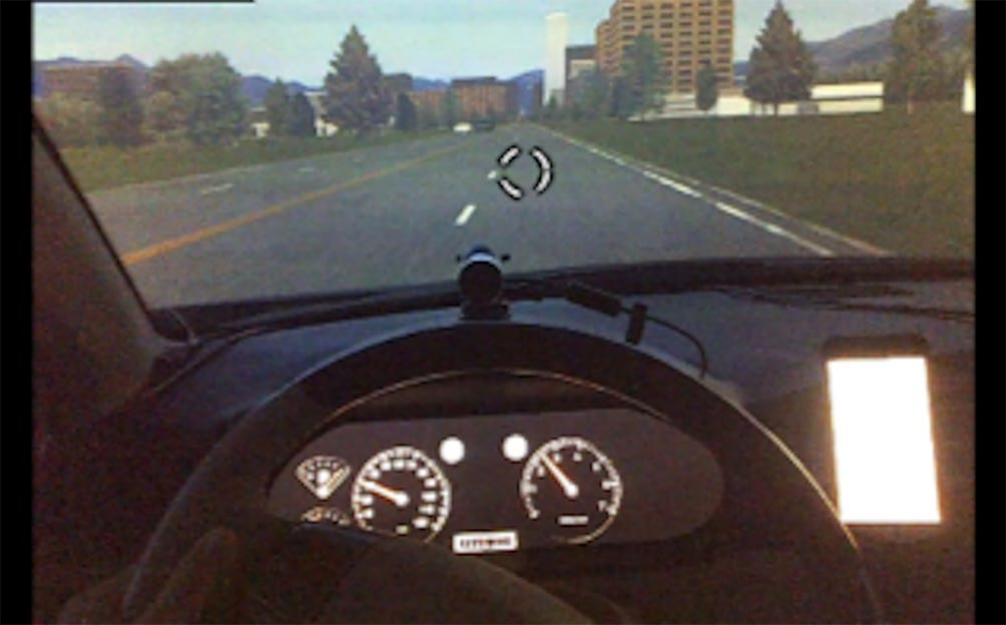
Single frame image from a participant’s eye-tracking video. The analysis software superimposed a fixation point (i.e., white circle on the road in this image) onto each video frame. For each frame the location of the participants fixation was manually coded as being on the road, phone, cab, or outside of the cab in a location other than the road. In the current image, the location would be coded as “road”. Coding of the videos in this manner allowed us to calculate the total duration viewed for each region, as well as viewing patterns during each of the distracting events.

First, the visual scene viewed by each participant while driving was categorized into four distinct sections, including the road, the phone, the inside of the cab, and outside of the cab on any location other than the road. Categorizations were made by manually reviewing all eye tracking videos captured by the eye tracking goggles. Specifically, participants’ gaze location was coded into one of the four regions for every video frame (see Fig. 5). The total proportion of time spent viewing each region was then calculated individually during each condition. Coding of all videos was distributed evenly across three authors (A.P., A.G., and L.K.H.), and inter-rater reliability was calculated on an overlapping subset of videos. Cohen’s kappa ($\widehat{\mu }= .912$) was calculated for all dual-coded videos. Next, a four (regions of interest) × five (conditions) repeated measures ANOVA was conducted. Follow-up pairwise comparisons were made using the Bonferroni method to correct for increased likelihood of Type I errors due to repeated testing.

#### Interaction analyses

In order to assess the relationship between each dependent measure category described above, we conducted a series of linear regression analyses using a forward stepwise variable entry method. For fNIRS and behavior metric analyses, the significant fNIRS clusters from the parametric analysis were used as independent variables (i.e., predictors) of each behavioral metric. Similarly, for the fNIRS and eye tracking analyses, the significant fNIRS clusters were used to predict gaze patterns. In both cases, it was assumed that cortical activity preceded, and thus influenced both driving and looking behavior. For the behavior and looking time analyses, the eye tracking data was used to predict driving behavior. Here, it was assumed that where the driver was looking would influence their driving behavior.

### Significance statement

As personal use technologies such as smartphones become ever more ubiquitous in today’s society, distracted driving related injuries and deaths are rising. While significant efforts have been made to increase the overall safety of modern automobiles, very little is currently understood about how drivers’ brains respond to smartphone interactions within naturalistic driving scenarios. Here, we provide evidence of parametrically modulated cortical brain activity that coincides with driving behavior as drivers engage naturally with common smartphone applications within an immersive virtual driving environment.

## Results

An alpha of 0.05 was used to assess statistical significance for all analyses reported below.

### Behavioral metrics

(see Table 1 for definitions).

#### Lateral acceleration

Lateral acceleration differed significantly across conditions, *F*(4,144) = 18.961, *MSE* = 0.024, *p* < 0.001, *η*^2^ = 0.107. Follow-up comparisons identified significantly lower lateral acceleration for open-ended text responding (OER) and GPS events compared to text banner, forced-choice text responding (FCR), and normal control driving.

#### Lateral jerk

Lateral jerk differed significantly across conditions, *F*(4,144) = 17.617, *MSE* = 0.001, *p* < 0.001, *η*^2^ = 0.434. Follow-up comparisons identified significantly lower lateral jerk for OER compared to FCR and CD, as well as between GPS and TB, FCR, and CD.

#### Heading error

Heading error differed significantly across conditions, *F*(4,144) = 3.733, *MSE* = 0.001, *p* = 0.006, *η*^2^ = 0.078, however, no follow-up comparisons for heading error were significant.

#### Lane excursion duration

Analysis of variance failed to reject the null hypothesis that mean lane excursion duration values differed across conditions, *p* > 0.05.

#### Lane drift

Lane drift differed significantly across conditions, *F*(4,144) = 50.155, *MSE* = 1.307, *p* < 0.001, *η*^2^ = 0.073. Follow-up comparisons indicated that lane drift was significantly lower for both OER and FCR compared to TB, GPS, and CD conditions.

#### Steering wheel angle

Steering wheel angle differed significantly across conditions, *F*(4,144) = 21.526, *MSE* = 0.002, *p* < 0.001, *η*^2^ = 0.398. Follow-up comparisons identified significantly lower average steering wheel angel for OER compared to TB and CD conditions, as well as between FCR and the TB conditions.

#### Steering wheel reversals

Steering wheel reversals differed significantly across conditions, F(4,144) = 6.894, MSE = 100.729, p < 0.001, *η*^2^ = 0.063. Follow-up comparisons indicated that wheel reversals were significantly lower during the OER and FCR conditions compared to the GPS and CD conditions.

#### Brake pedal force

Brake force values differed significantly across conditions, *F*(4,144) = 35.446, *MSE* = 15.024, *p* < 0.001, *η*^2^ = 0.361. Follow-up comparisons indicated that brake force was greater during the GPS condition compared to all others.

### fNIRS metrics

#### Parametric analysis

**Figure 6:**
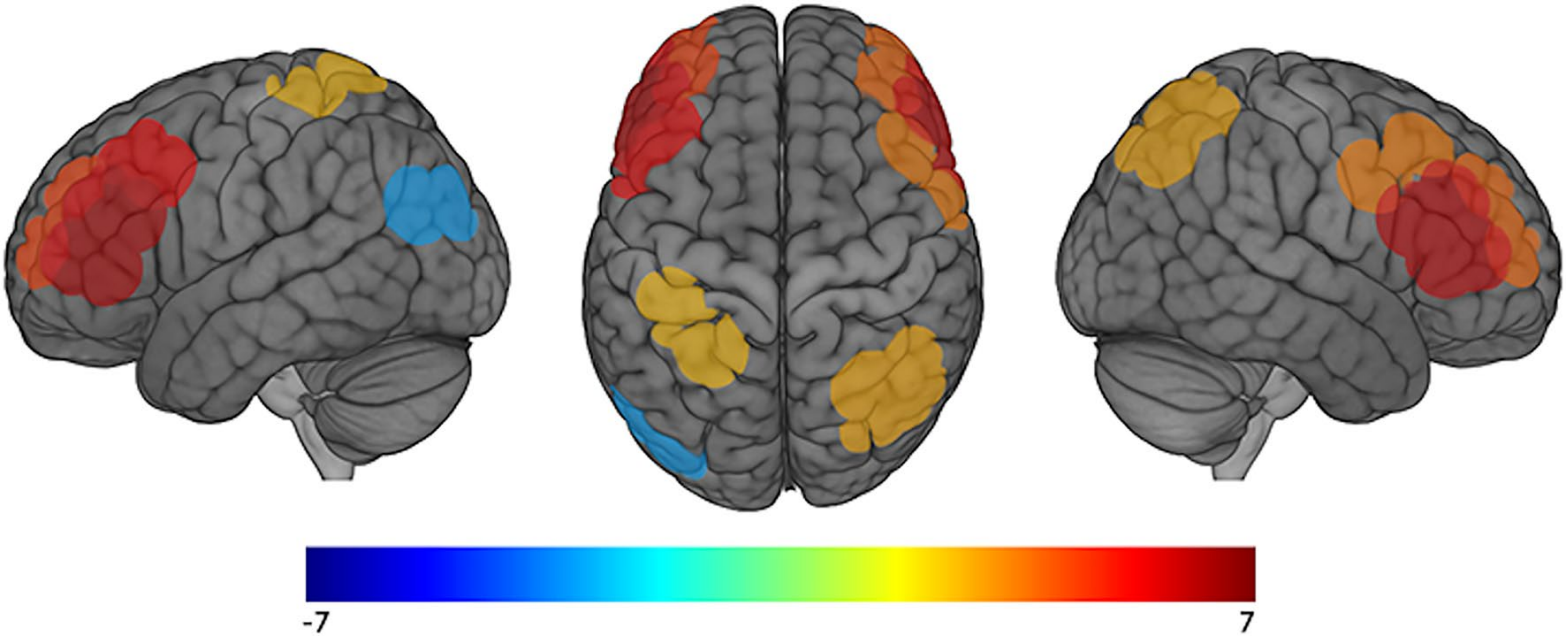
fNIRS parametric GLM analysis outcomes. Conditions were weighted parametrically based on their a priori level of distraction. Weighting was as follows: Standard driving < Active GPS alert < Text banner alert < FCR response < OER response. Hot colors indicate positive correspondence of cortical activity to the weighted condition order; Cold colors indicate inverse correspondence of cortical activity to weighted condition order. Thus, bilateral prefrontal activity increased from GPS to text response conditions, whereas left inferior parietal activity was greatest for GPS alerts and decreased in response to text conditions.

A parametric GLM analysis identified a positive association with increased condition-related distraction in regions of the left pre-frontal and parietal cortices. The significant (FDR *p* < 0.05 for all t-statistics shown) left prefrontal regions of interest included the left inferior frontal pole (S2, *t* = 5.195, Cohen’s *d* = 1.46), the left dorsolateral PFC (S3, *t* = 6.171, Cohen’s *d* = 1.75), and the left ventrolateral PFC (S4 , *t* = 7.254, Cohen’s *d* = 2.05). The significant right prefrontal regions of interest included the right ventrolateral PFC (S5, *t* = 7.049, Cohen’s *d* = 1.99), the right inferior frontal pole (S6, *t* = 4.500, Cohen’s *d* = 1.27), and the right dorsolateral PFC (S7, *t* = 4.116, Cohen’s *d* = 1.16). Furthermore, positive associations were identified in the left superior parietal (S9, *t* = 3.050, Cohen’s *d* = 0.86) and right central parietal (S15, *t* = 3.338, Cohen’s *d* = 0.94) cortices. A negative association with increased parametric modulation was observed in the left temporo-parietal junction, source cluster S12 (*t* = − 3.617, Cohen’s *d* = 1.04) (Fig. 6).

#### Condition contrast analysis

**Figure 7:**
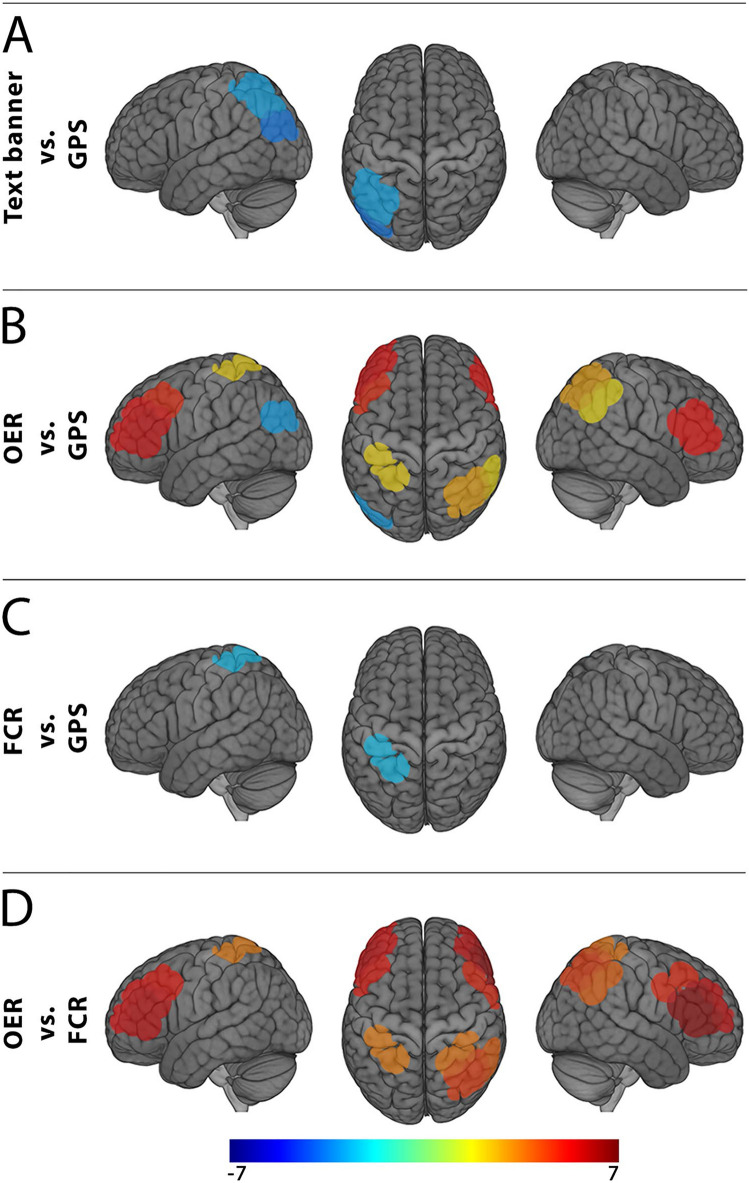
fNIRS contrast outcomes. (**A**) Text banners were identical for both OER and FCR events. Presentation of the text banner failed to elicit significantly greater prefrontal activity compared to GPS events. However, GPS events elicited greater activity in the left central and left temporo-parietal junction compared to text banner alerts. (**B**) Drivers responding to OER text events elicited greater activation in the left inferior frontal pole, left dorsolateral PFC, bilateral HELP, right angular gyrus, right central parietal, and left superior parietal cortices. Activity in the left temporo-parietal junction was greater for GPS compared to OER events. (**C**) FCR events failed to elicit the same patterns of cortical activity, while cortical activity in the left superior parietal cortices remained significantly higher for GPS compared to FCR events. (**D**) Comparison of OER and FCR responses identified significantly greater cortical activity during OER events within the bilateral inferior frontal pole, bilateral dorsolateral PFC, bilateral HELP, right angular gyrus, right central parietal, and the bilateral superior parietal cortices.

T-test analyses indicate greater cortical response in the left central parietal (S10, t = − 3.247, Cohen’s *d* = 0.92) and left temporo-parietal junction (S12, t = − 4.211, Cohen’s *d* = 1.19) regions of interest in response to GPS compared to text banner events (see Fig. 7A). Next, contrasts made between cortical activations during participants’ responses to open-ended texting compared to receiving GPS information indicated greater activity for texting within the left inferior frontal pole (S2, t = 6.114, Cohen’s *d* = 1.73), left dorsolateral prefrontal (S3, t = 5.532, Cohen’s *d* = 1.56), the left ventrolateral prefrontal (S4, t = 6.462, Cohen’s *d* = 1.83), right ventrolateral (S5, t = 6.012, Cohen’s *d* = 1.70), right angular gyrus (S13, t = 2.358, Cohen’s *d* = 0.67), right central parietal (S15, t = 3.342, Cohen’s *d* = 0.95), and left superior parietal (S9, t = 2.498, Cohen’s *d* = 0.71) cortices. Furthermore, cortical activity during these conditions was greater for GPS information relative to OER within the left temporo-parietal junction (S12, t = − 3.455, Cohen’s *d* = 0.98) (see Fig. 7B). Next, contrasts made between the FCR and GPS conditions indicated greater left superior parietal (S9, t = − 2.832, Cohen’s *d* = 0.80) activity in response to GPS (see Fig. 7C). Finally, contrasts made between OER and FCR conditions indicated that cortical activity was greater for OER in the left inferior frontal pole (S2, t = 6.250, Cohen’s *d* = 1.77), left dorsolateral prefrontal (S3, t = 5.860, Cohen’s *d* = 1.66), left ventrolateral (S4, t = 6.711, Cohen’s *d* = 1.90), right ventrolateral (S5, t = 7.692, Cohen’s *d* = 2.18), right inferior frontal pole (S6, t = 6.639, Cohen’s *d* = 1.88), right dorsolateral prefrontal (S7, t = 5.589, Cohen’s *d* = 1.58), right angular gyrus (S13, t = 4.547, Cohen’s *d* = 1.29), right central parietal (S15, t = 5.238, Cohen’s *d* = 1.48), right superior parietal (s16, t = 4.172, Cohen’s *d* = 1.18), and left superior parietal (S9, t = 4.048, Cohen’s *d* = 1.14) regions (see Fig. 7D).

### Eye tracking analysis

**Figure 8:**
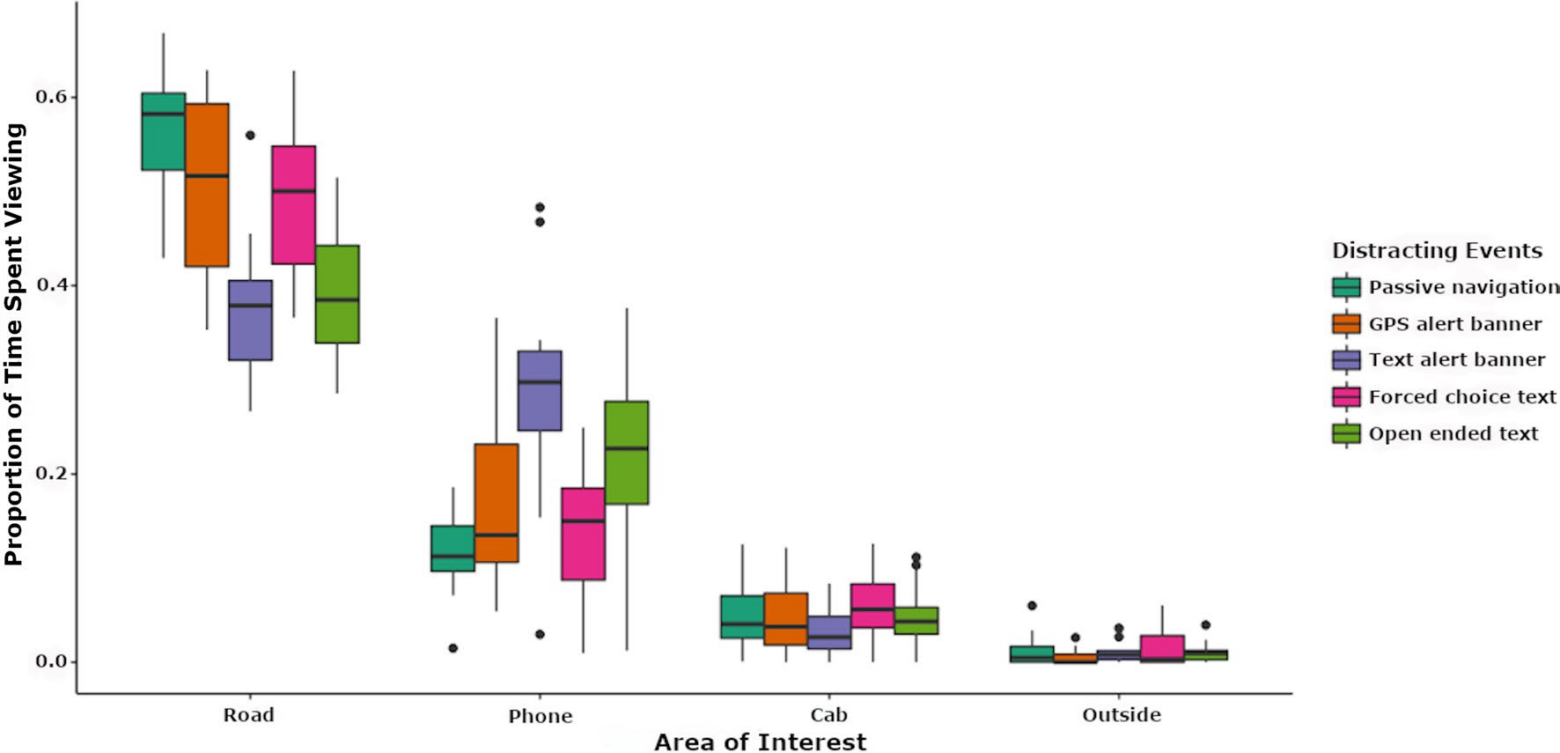
Eye tracking outcomes. The proportion of time each driver spent viewing each location differed. Fortunately, drivers spent the greatest proportion of time viewing the road, followed by the phone, cab, and finally outside of the car but not on the road. A significant location x activity interaction is driven by an abrupt relative change in viewing proportions of the TB and OET conditions between the road and phone locations. This is evident by these conditions having the lowest median proportions within the road location (i.e., drivers spent relatively little time looking at the road during these conditions) but having the highest proportion within the phone location (i.e., drivers viewed the phone relatively longer during these conditions).

A five (smartphone event conditions) x four (eye tracking locations of interest) repeated measures ANOVA identified a significant location x activity interaction (*F*(4, 340) = 18.69, *MSE* = 0.065, *p* < 0.001, Partial *η*^2^ = 0.397), driven by relatively low proportion of time spent viewing the road during TB and OET events, along with greater proportion of time spent viewing the phone during these events. This interaction was supported by a significant main effect of location (*F*(3, 340) = 1099.618, *MSE* = 3.839, *p* < 0.001, Partial *η*^2^ = 0.907; see Fig. 8 for distribution of eye tracking patterns). Follow-up pairwise comparisons identified significantly greater looking (FDR *p* < 0.05) time proportions to the road compared to the phone, cab, and outside regions of interest. This comparison also identified significantly greater looking time proportions for the phone compared to the cab, and outside regions of interest. Participants also spent significantly more time viewing regions of the cab compared to outside regions of interest. No other comparisons were significant.

### Outcome metric interactions

#### fNIRS and behavior

We employed stepwise regression to assess the relationship between each significant behavioral metric and the patterns of cortical activity associated with parametric changes in distraction within the brain regions reported above. This analysis identified a significant negative relationship between heading error during open-ended responding and activity in the right ventrolateral region (S5, *F* = 5.134, *b*_*1*_ = − 0.0004), as well as a negative relationship between steering wheel angle during text banner alerts and activity in the left dorsolateral prefrontal region (S3, *F* = 4.887, *b*_*1*_ = − 0.004). A positive relationship was identified between brake force during GPS banner alerts and activity in the left temporal-parietal junction (S12, *F* = 5.432, *b*_*1*_ = 0.018). Moreover, parametrically modulated cortical activity in the left temporo-parietal junction (S12) and right central parietal cortices (S15) was significantly related to driver’s brake force during GPS alert banners (*F* = 8.238, *b*_*1*_ = 0.043, *b*_*2*_ = − 0.028). Finally, lane excursion during open-ended responding was significantly predicted by a combination of activity in the left inferior prefrontal cortex (S2) and right central parietal regions (S15, *F* = 7.503, *b*_*1*_ = − 0.066, *b*_*2*_ = 0.038).

#### fNIRS and NEO interaction

There were no significant relationships identified between parametrically modulated cortical activity and NEO outcomes.

#### fNIRS and BRIEF interaction

Regression analysis identified a significant positive relationship between parametrically modulated activity in the left temporo-parietal junction (s12) and the Plan to Organize (*F* = 4.680, *b*_*1*_ = 0.306) and Task Monitor (*F* = 7.507, *b*_*1*_ = 0.115) scales. Furthermore, the Organization of Materials scale was positively related to parametrically modulated activity in the same parietal region, while being negatively related to activity in the left ventrolateral (s4, *F* = 7.339, *b*_*1*_ = 0.144, *b*_*2*_ = − 0.102).

#### Behavior and NEO interactions

This analysis identified a significant negative relationship between participants’ brake force when they receive a GPS alert banner and Conscientiousness (*F* = 4.494, *b*_*1*_ = − 0.039). Conscientiousness was also significantly related to brake force during text banner alerts (*F* = 7.399, *b*_*1*_ = − 0.026). Moreover, participants Agreeableness scores were significantly related to lane excursion during text banner alerts (*F* = 5.621, *b*_*1*_ = − 0.040), as well as lane excursion during forced choice responding (*F* = 4.628, *b*_*1*_ = − 0.065). Finally, lane drift during forced-choice responding was significantly related to participant’s Neuroticism score (*F* = 7.246, *b*_*1*_ = 0.003).

#### Behavior and BRIEF interactions

This analysis indicated that Lateral Jerk, when participants received a text alert banner, was significantly predicted by a combination of Initiate and Self Monitor scales (*F* = 5.890, *b*_*1*_ = − 2.918, *b*_*2*_ = 0.001). Moreover, Lateral Jerk during open-ended text responding was significantly predicted by a combination of Emotional Control and Initiate scales (*F* = 8.359, *b*_*1*_ = 0.001, *b*_*2*_ = − 0.001). Heading error during open-ended responding was significantly predicted by a combination of Initiate and Inhibit scales (*F* = 4.766, *b*_*1*_ = − 0.002, *b*_*2*_ = 0.002). Participant’s Lane Drift during text alert banners was negatively related to the Initiate scale (*F* = 6.627, *b*_*1*_ = − 0.035). Furthermore, Lane Drift during open-ended text responding was significantly predicted by a combination of Initiate and Inhibit scale (*F* = 5.321, b1 = − 0.011, *b*_*2*_ = 0.110).

#### fNIRS metrics and looking-time proportion interactions

There were no significant relationships identified between parametrically modulated cortical activity and looking time proportions.

#### Behavioral metrics and looking-time proportion interactions

There were no significant relationships identified between behavioral metrics and looking time proportions.

## Discussion

Our study provides a unique insight into the neural and behavioral interplay while drivers experience common forms of smartphone distraction. Importantly, our innovative methodology allowed us to capture different aspects of drivers’ response to distracting events, within a simulated environment that closely mimicked the real world. Furthermore, our use of GPS- and text-related interactions allowed us to test the effects of different degrees of driver distraction in a manner that closely approximated smartphone interactions that today’s drivers commonly experience.

Our parametric analysis indicated that drivers recruit significantly greater cortical activity throughout the bilateral PFC, right central parietal, and left superior parietal regions as the level of smartphone distraction increased from GPS to OER events (see Fig. 6). Conversely, this analysis indicated that the left temporo-parietal junction responded greatest to GPS events and became less active as distraction increased. These results were complimented by our contrast analyses, which highlights similar patterns of activations for OER and GPS events. That is, the bilateral PFC, right central parietal, and left superior parietal regions responded greatest to OER events in each relevant contrast (Fig. 6b,d), whereas each contrast containing GPS events indicated greater cortical activation throughout the left parietal regions (Fig. 7A-C). Notably, the conditions that constituted mid-range distracting events (e.g., text alert banner and FCR texts) did not elicit cortical activation in their favor in our contrast analyses. In other words, only the conditions on the extreme ends of our distraction spectrum elicited patterns of activation over-and-above other conditions, and those patterns of activation did not overlap. We interpret these results as justification for our parametric weighting of each distraction condition.

Our results support previous findings that demonstrate a shift in cortical activity to the bilateral PFC in response to distracted driving^16^. These findings may be expected, as prefrontal regions are known to underlie cognitive processes related to attention^45^, working memory^46,47^, and problem solving^48^—each of which is necessary to handle increased levels of distraction and likely occur simultaneously during moments of distracted driving. Furthermore, our results also highlight the left temporo-parietal junction and other regions throughout the left parietal region as being significantly active during GPS events. Notably, cortical activity in the left temporo-parietal junction has been shown to underlie meta-cognitive processes such as the collection and processing of information from inside and outside of the body^49^, and perspective taking^50^. Thus, our findings may indicate that GPS feedback evokes cortical responses that help the driver orient themselves in their surrounding environment, but do not require the relatively high levels of working memory or attentional resources demanded by highly distracting events (i.e., texting).

Along with changes in cortical activation, our distraction manipulations also influenced driving behavior. Specifically, highly distracting events tended to lessen the magnitude of each behavioral metric of interest. For example, many of our behavioral metrics are derived from the driver’s steering wheel input (e.g., lateral jerk, wheel reversal, etc.), and are relatively high under normal driving conditions due to the driver’s manipulation of the wheel to correct or maintain course. The tendency for many of these metrics to decrease significantly during highly distracting events (e.g., OER) compared to less distracting events indicates that participants interacted with the steering wheel less when they were distracted. Meanwhile, our eye tracking results indicate that drivers removed their eyes from the road and focused more on the smartphone during OER and text banner alerts events compared to other conditions. Interestingly, many of the cortical activations identified during OER and GPS events in our study significantly predicted the changes we observed in drivers’ behavior. Taken together, our results highlight multiple neurobiological signatures of distracted driving that significantly influence drivers’ behavior.

Our results may have important implications for the development of non-invasive physiological monitoring tools that can be used to assess drivers’ attention. For example, monitoring of drivers’ eyes, including their blink patterns over time, has been shown to capture driver fatigue^51^. Furthermore, increases in task demands have been shown to produce attentional focus narrowing (i.e., spatial gaze concentration), which manifests as a fixated gaze on a single object at the attentional expense of visual objects in the viewers periphery^52^. When conducted in more naturalistic on-road studies, research shows that the influence of distraction on gaze patterns was independent of the location of the visual distraction^53^, suggesting that cognitive tunneling may not be present in real-world driving and that increased distraction may result in a more general visual interference effect. These results may be difficult to reconcile with ours, in large part because of differences between study designs. That is, the nature of our distracting tasks required the participant to view a specific region of their visual field (i.e., the phone), and increased distraction (i.e., GPS alert banner to open-ended texting) inherently required additional time to complete. It is feasible that fixation patterns within each region of interest deviated across distracting conditions, although this analysis is out of the scope of our study. We encourage future researchers to address this question directly.

Importantly, as our results demonstrate, neurobiological signatures of distraction that influence driver behavior may be captured by fNIRS. Given its low cost, ease of setup, and robust tolerance to the driving environment, fNIRS may provide a viable tool to monitor driver attention in the real world. Future research is needed to replicate and extend our results with the aim of identifying the minimum number of channels needed to monitor driver attention so that the size and cost of in-vehicle systems may be reduced as much as possible. Importantly, such research should be conducted in collaboration with engineers and human factors experts so that a viable form factor and application method may also be developed.

## Limitation and future directions

It is possible that our observed patterns of behavioral metric dampening are a result of the simulated environment in which participants were driving. For example, the roads in the simulated environment lacked the camber (i.e., crowning that causes a slightly arched road surface) that is common on real roads. The physical effect of road camber is unintended lateral movement of the automobile, which must be corrected by steering wheel inputs. Similarly, our simulated environment was devoid of wind, which also causes unexpected and abrupt lateral movement of an automobile. As a result, in our study, when participants removed their hands from the steering wheel the car would travel in a straight line until steering wheel input was applied. Thus, participants may have used this inherent tendency when texting, resulting in the decrease that we observed. Conversely, similar driving behavior conducted in more naturalistic environments likely causes drastic movements of the automobile along the road, and thus more dangerous scenarios. Future research is needed to further probe the neurobiological signatures we have identified above in environments that provide even greater fidelity in such real-world aspects of driving.

In our study, we employed wide variety of behavioral metrics that describe how the automobile moved through the virtual environment, as well as how the driver interacted with the vehicle via the steering wheel and brake pedal (see Table 1). Many of the behavioral variables provided complementary yet unique information about the driving experience. For instance, a driver may employ multiple strategies to drive the automobile down the center of a lane with minimal lateral drift. First, one driver may keep the steering wheel relatively still, making minimal corrections and consequently minimizing abrupt movements along the lateral axis (e.g., lateral acceleration and jerk). Alternatively, another driver may continuously adjust the steering wheel, thereby maintaining minimal variation at the expense of higher lateral motion. In these examples, we may expect many variables (e.g., lane drift, heading error, lane excursion) to be similar between the two drivers, although metrics related to driver input (e.g., steering wheel reversals) may vary. We argue that it is feasible that such differences in driving style may coincide with variations in neural, behavioral, or neuropsychological signatures of each driver. Given the novel and exploratory nature of our study, we chose to include each variable for analysis. However, future studies may aim to interrogate individual behavioral driving metrics more closely in relation to neural and physiological signatures of distracted driving.
